# A Machine Learning based model for a Dose Point Kernel calculation

**DOI:** 10.21203/rs.3.rs-2419706/v1

**Published:** 2023-01-09

**Authors:** Ignacio Scarinci, Mauro Valente, Pedro Pérez

**Affiliations:** 1 Instituto de Física Enrique Gaviola (IFEG), CONICET, Av. Medina Allende s/n, Córdoba, 5000, Córdoba, Argentina.; 2 Laboratorio de Investigación e Instrumentación en Física Aplicada a la Medicina e Imágenes de Rayos X (LIIFAMIRx), Facultad de Matemática, Astronomía, Física y Computación, Universidad Nacional de Córdoba, Av. Medina Allende s/n,, Córdoba, 5000, Córdoba, Argentina.; 3 Centro de Excelencia en Física e Ingeniería en Salud (CFIS) & Departamento de Ciencias Físicas, Universidad de la Frontera, Avenida Francisco Salazar 01145, Temuco, 4811230, Cautín, Chile.

**Keywords:** BETA EMITTERS, DOSE POINT KERNEL, INTERNAL DOSIMETRY, MACHINE LEARNING

## Abstract

**Purpose:**

Absorbed dose calculation by kernel convolution requires the prior determination of dose point kernels (DPK). This study shows applications of machine learning to generate the DPKs for monoenergetic sources and a model to obtain DPKs for beta emitters.

**Methods:**

DPK for monoenergetic electron sources were calculated using the FLUKA Monte Carlo (MC) code for many materials of clinical interest and initial energies ranging from 10 to 3000 keV. Three machine learning (ML) algorithms were trained using the MC DPKs. Electron monoenergetic scaled DPKs (sDPKs) were used to assess the corresponding sDPKs for beta emitters typically used in nuclear medicine, which were compared against reference published data. Finally, the ML sDPK approach was applied to a patient-specific case calculating the dose voxel kernels (DVK) for a hepatic radioembolization treatment with ^**90**^Y.

**Results:**

The three trained machine learning models demonstrated a promising capacity to predict the sDPK for both monoenergetic emissions and beta emitters of clinical interest attaining differences lower than **10%** in the mean average percentage error (MAPE) as compared with previous studies. Furthermore, differences lower than **7%** were obtained for the absorbed dose in patient-specific dosimetry comparing against full stochastic MC calculations.

**Conclusion:**

An ML model was developed to assess dosimetry calculations in nuclear medicine. The implemented approach has shown the capacity to accurately predict the sDPK for monoenergetic beta sources in a wide range of energy in different materials. The ML model to calculate the sDPK for beta-emitting radionuclides allowed to obtain VDK useful to achieve reliable patient-specific absorbed dose distributions required remarkable short computation times.

## 1 Introduction

Personalized medicine advances have significantly enhanced the efficacy of therapeutic and palliative treatments for several diseases [1–3]. The introduction of theragnostic therapies, combining therapeutic and diagnostic imaging using a single radiopharmaceutical, has increased interest in radiopharmaceuticals in various cancer treatments, particularly in nuclear medicine [4–6]. The capacity to generate molecular imaging, such as SPECT or PET, used for treatment procedures for real-time monitoring of the whole radiopharmaceutical metabolization process, together with the capacity to obtain anatomical images alongside molecular imaging (SPECT/CT, PET/CT, PET/MRI), allows for significant improvements in dosimetric estimates both before and after treatment [7–9]. Thus, theragnostic enables patient-specific dosimetric calculations based on molecular and anatomical imaging, improving radionuclide treatment effectiveness and safety [10, 11].

Several approaches for internal dose estimation in nuclear medicine procedures have been developed, such as Monte Carlo (MC) transport simulation [12, 13], S-value estimation [14, 15], and dose point kernel (DPK) convolution [16, 17]. The MC method is the most precise dosimetric calculation approach, but it requires many computational resources and long computation times, making it sometimes inappropriate for clinical usage. On the other hand, methods such as S-values or DPK convolution allow for shorter computational times at the expense of lower computational accuracy. Thus, a tradeoff between computational time and the precision or accuracy of the dosimetric calculation is desirable.

Calculating the absorbed dose by convolution of DPKs requires a prior calculation of the DPKs. An extensive list of beta DPKs has been published [18–23]. Also, different methods for scaling DPKs to different media have been propose since they are commonly calculated for water; then, DPKs for other media are obtained by applying different scaling factors [24, 25]. From the calculated beta DPKs, the so-called Voxel S-values (VSV) or Voxel Dose Kernel (VDK) can be obtained, which facilitate the absorbed dose calculation by convolution with the accumulated activity since they are represented in the form of three-dimensional matrices, and the convolution product can be solved in a discrete form, simplifying the calculation of the absorbed dose [26].

Artificial intelligence (AI) disrupted many fields of medical sciences due to computational advances that occurred over the last decade in terms of innovative hardware and software [27, 28], also impacting nuclear medicine [29–31]. In the internal dosimetry field, some Deep Learning (DL) based models have been developed to predict patient-specific doses based on anatomic and metabolic imaging information [32–34].

## 2 Methods and Materials

### 2.1 Radionuclides and materials

Table 1 resumes some beta-emitting radionuclides usually applied in treating different diseases [35–39]. The overall maximum energy emitted is 2275.6*KeV* for ^90^Y; therefore, energy values between 10 and 3000 *keV* were considered for monoenergetic beta sDPK calculation, whereas a method for calculating the sDPK for a spectral emission of beta particles from the sDPK for a monoenergetic beta emission is depicted in the following section.

As usual, the studied materials were divided into two sets: the training and the testing sets. Material information in terms of Hounsfield Unit (HU), as described by Schneider et al. [40], was used to define the training set composition, and mass densities were assessed using the mean value of the HU considered range [41]. Subsequently, the following materials were used as testing compositions: air, lung, soft tissue, and cortical bone, according to the ICRP Publication 89 [42]. Table 2 and 3 resume the composition of the training and testing set.

### 2.2 Beta-emitting radionuclide sDPK

The electron-beta DPK is a function that represents the radial distribution of a specific absorbed fraction of dose in an infinite homogeneous medium due to a monoenergetic point source of beta or electron particles. A more useful form of DPK is the scaled sDPK, defined as

(1) $$F\left(\frac{r}{r_{0}}\right)=4\pi r^{2}r_{0}\text{Φ}(r)$$

where *ρ* is the medium’s density and *r*_0_ the range in the Continuous Slowing Down Approximation (*R*_*CSDA*_) approximation. The beta-emitting radionuclide sDPK can be defined as

(2) $$F_{\beta }\left(\frac{r}{r_{N}}\right)=4\pi r^{2}r_{N}{\text{Φ}}_{\beta }(r)$$

where *r*_*N*_ is the range for the maximum emission energy of the radioisotope, Φ_*β*_(*r*) is the fraction of absorbed energy at a distance *r* [24]. Considering an infinite sphere of a homogeneous material with a source of beta particles in the center, the fraction of absorbed energy at a distance from the center is defined as [43]

(3) $${\text{Φ}}_{\beta }(r)=\frac{\int_{0}^{E_{\max}}E_{0}\frac{dI}{dE_{0}}\text{Φ}\left(r,\;E_{0}\right)\;dE_{0}}{\int_{0}^{E_{\max}}E\frac{dI}{dE_{0}}dE_{0}}$$

and Φ_*β*_(*r, E*) is the fraction of absorbed energy at a distance *r* to energy *E*. Equation 3 is approximated by

(4) $${\text{Φ}}_{\beta }(r)=\frac{\sum_{j}I_{j}E_{0j}{\text{Φ}}_{j}\left(r,\;E_{0j}\right)}{E_{eff}}$$

where *I*_*j*_ is the strength of the j-th group with mean energy *E*_0*j*_, Φ(*r, E*_0*j*_) is the fraction of absorbed energy at a distance due to the j-th group of the spectrum and the effective energy is $E_{e}ff=\sum_{j}I_{j}E_{0j}$. From equation 2 and equation 4,

(5) $$F\left(\frac{r}{r_{N}}\right)=\frac{\sum_{j}^{4}\pi \;\rho \;r^{2}r_{N}\;I_{j}\;E_{0j}\;{\text{Φ}}_{j}\;\left(r,\;E_{0j}\right)}{E_{eff}}$$

Introducing equation 1 in 5, the sDPK of a betta-emitting radionuclide can be estimated from the sDPK for monoenergetic electron source as

(6) $$F\left(\frac{r}{r_{N}}\right)=\frac{r_{N\sum_{j}I_{j}}E_{0j}\frac{F_{j}\left(\frac{r}{r_{0j}},E_{j}\right)}{r_{0j}}}{E_{eff}}$$

Equation 6 states that knowing the sDPK for the monoenergetic source and the emission spectra of the radionuclide are required to obtain the sDPK for the radionuclide.

### 2.3 DPK estimation by Monte Carlo simulations

The DPK for each material was obtained through MC simulations using the general-purpose software FLUKA version 2021.2.0, capable of calculating detailed radiation transport and energy deposition [44, 45]. FLUKA can simulate the whole track of several particles like photons, electrons, neutrons, and hadrons on a wide range of energies. It has been widely used for high-energy physics, experiencing an increasing application for medical physics purposes [46, 47]. FLUKA implements an original algorithm for treating multiple scattering on charge particles transport based on the Bethe improved Moliere’s theory [48].

FLUKA incorporates standard configurations which activate/deactivate by default various features according to the required physical model; in this study, the PRECISIOn default was applied, activating the electromagnetic interactions, the Rayleigh scattering, and the inelastic form factor corrections to Compton scattering and Compton profiles. The transport and production threshold was set on 1 keV for electrons and photons with initial energy under 100 keV, and it was set on 10 keV energies above 100 keV. Furthermore, single scattering was set up at boundaries for electron energies from 10 to 100 keV. Preliminary tests showed that 10 independent cycles of 10^6^ primary particles each cycle were the appropriate configuration to be set to obtain accurate results.

The phantom used for DPK calculations consists of 60 concentric spherical shells of homogeneous material whose outer radius is 1.5*R*_*csda*_. Each shell has a thickness of *R*_*CSDA*_/40. The *R*_*CSDA*_ value is obtained using the fitting proposed by Tabata *et al.* [49], where the *R*_*CSDA*_ is calculated taking into account the effective atomic number *Z*_*eff*_ and the effective atomic weight *A*_*eff*_ of compound. Also, a monoenergetic electron source was positioned at the center of the spheres.

FLUKA provides the deposited energy *δE* on each *δr* thick shell. It is convenient to define the sDPK according to the results obtained from simulations and the initial kinetic energy *E*_0_ expressed in MeV, and the range *R*_*CSDA*_ expressed in cm as [21]

(7) $$F\left(\frac{r}{R_{CSDA}}\right)=\frac{\delta E(r)/E_{0}}{\delta r/R_{CSDA}}$$

### 2.4 sDPK estimation by ML

The problem of predicting monoenergetic sDPK from physical and chemical properties can be represented by a multivariate or multi-target regression [50]. Let *D* be a training dataset made up of *N* instances such that *D* = (*X*_1_, *Y*_1_),...,(*X*_*N*_*, Y*_*N*_). Likewise, each sample consists of an input array *X*_*N*_ of dimensions *m* such that *X*_*i*_ = (*x*_1_, ..., *x*_*m*_) and a target array *Y* of sizes *k* such that *Y*_*i*_ = (*y*_1_, ..., *y*_*m*_). The problem is reduced to training a multi-target regressor model, which consists of finding a function *h* that assigns an array *Y* to each array *X*, that is

(8) $$Y=h(X):h:{\mathbb{R}}^{m}\to {\mathbb{R}}^{k}$$

The algorithm used was an ensemble of regressor chains [51]; this ensemble *M* was composed of *k* base regressors *m*, so that *M*(*m*) = [*M*_1_(*m*)*, ..., M*_*i*_(*m*), ...*M*_*k*_(*m*)]. The dimension *k* is the same as the dimension of the target array *Y*. Firstly, a model *M*1 is trained with all input features *X* and the first element of the target array *y*_1_, then, a second model *M*_2_ is trained with elements of *X* along with the first element *y*_1_ as input features, and the second element of *Y* array *y*_2_ is the target. Repeat this procedure until all *M* models for each element of array *Y* have been trained.

Three base regressors were studied: Ridge [52], Lasso [53], and Elastic Net [54] which use different regularized terms. The Ridge algorithm minimizes the residual sum of squares subject to bound on the L2-norm of the coefficients:

(9) $$\underset{w}{\min}∥Xw-y{∥}_{2}^{2}\;+\;\alpha ∥w{∥}_{2}^{2}$$

where *α* ⩾ 0 is a constant, and $∥w{∥}_{2}^{2}$ is the L2-norm of the coefficient vector. The Lasso algorithm is a penalized least-squares method imposing an L1-penalty on the regression coefficients:

(10) $$\underset{w}{\min}∥Xw-y{∥}_{2}^{2}\;+\;\alpha ∥w∥$$

where *α* is a constant, and ∥*w*∥_1_ is the L2-norm of the coefficient vector. The Elastic Net algorithm penalized the least-squares method using a combination of both kinds of regularization:

(11) $$\underset{w}{\min}\frac{1}{2n_{samples}}∥Xw-y{∥}_{2}^{2}\;+\;\gamma \alpha ∥w{∥}_{1}\;+\;\frac{\alpha (1\;-\;\gamma )}{2}∥w{∥}_{2}^{2}$$

where *α* and *γ* are constants, ∥*w*∥_1_ and $∥w{∥}_{2}^{2}$ are the L1-norm and L2-norm of the coefficients vector, respectively.

The system was characterized by the following features of the source: energy *E*_0_[*keV*], the range *R*_*CSDA*_[*g/cm*^2^], density of medium material *ρ*[*g/cm*^3^], and the composition of the material in weight fraction for the elements considered (H, C, Na, Mg, P, S, Cl, Ar, K, Ca) and the monoenergetic sDPK was the target.

Two metrics were used to evaluate the models were the coefficient of determination (*R*^2^) and the Root Mean Square Error (RMSE), defined as

(12) $$R^{2}\left(y,y^{\prime }\right)=1\;-\;\frac{\sum_{i=1}^{n}{\left(y_{i}\;-\;y_{i}^{\prime }\right)}^{2}}{\sum_{i=1}^{n}{\left(y_{i}-{\bar{y}}_{i}\right)}^{2}}\;RMSE\left(y,\;\;y^{\prime }\right)=\sqrt{\frac{1}{n_{samples}}\sum_{i=0}^{n_{samples}-1}{\left(y_{i}-y_{i}^{\prime }\right)}^{2}}$$

where *y*_*i*_ and $y_{i}^{'}$ are i-th components of the sDPK calculated by MC and ML models, respectively.

The model predicts for a given energy and chemical composition of the medium the monoenergetic sDPK and applies equation 6 to obtain the sDPK for a given beta emitter in a particular medium.

### 2.5 Benchmark evaluation of the calculated sDPK for beta-emitting radionuclides

Beta-emitting radionuclide sDPK calculated by equation 6 was benchmarked against previously reported by Botta et al. [55] and Shiiba et al. [56]. Botta used FLUKA for simulating the sDPK, and Shiiba used PHITS (56). The radionuclides considered were ^89^Sr, ^90^Y, ^131^I, ^177^Lu, ^186^Re, and ^188^Re; the materials considered were water and compact bone. The Mean Absolute Percentage Error (MAPE) was used as a metric, defined as

(13) $$MAPE\left(F,\;F^{\prime }\right)=\frac{100}{n_{samples}}\sum_{i=1}^{n_{shells}}\frac{\left\|F_{i}\;-\;F_{i}^{\prime }\right\|}{\left\|F_{i}\right\|}$$

where *F* was the reference sDPK and *F*′ the sDPK estimated by the ML model for the i-th shell and *n* is the number of samples.

### 2.6 Dosimetry calculation

**Fig. 1:**
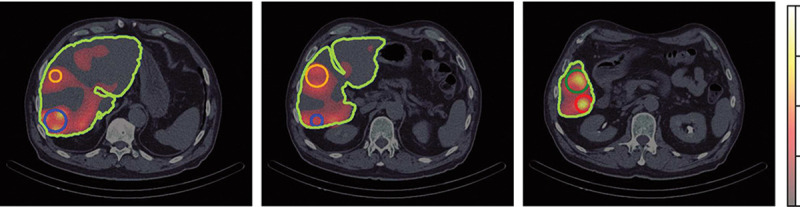
Three axial slices of fusion SPECT/CT with Liver and VOIs consider contoured

The SPECT/CT image obtained from Technetium ^99*m*^Tc albumin aggregated (^99*m*^Tc-MAA) pretreatment simulation before ^90^Y hepatic radioembolization was used to calculate the absorbed dose by applying FLUKA MC and Voxel Kernel Convolution (VKC) [26]. Figure 1 shows three axial slices of the image of a patient who was administered 185 *MBq* of ^99*m*^Tc-MAA. The image size was 512×512×258 pixels and a resolution of 0.98×0.98×0.98*mm*^3^. Also, images show the segmentation of the liver and 5 VOIs. Biodistribution of ^99*m*^Tc-MAA and ^90^Y microsphere were considered identical.

A source routine was developed to perform the MC simulation of the absorbed dose through FLUKA introducing from external file information the position of the active voxel and the number of primary particles to be simulated proportional to the number of counts in the voxel. One hundred independent cycles of 10^8^ primary particles were simulated to achieve an acceptable level of statistical error. The CT images were transformed into a voxelized phantom to convert the HU number to material composition and mass density by calibration [40].

Monoenergetic electrons sDPK have been calculated by ML model, and then equation 6 was applied to obtain ^90^Y sDPK. A 23×23×23 voxelized kernel with 1 mm3 pixel size was calculated using MC volume integration of ^90^Y sDPK [57]. The activity map was obtained from a ^99*m*^Tc-MAA image using the equation [58]

(14) $$A_{voxel}\left({}^{90}Y\right)=\frac{A_{liver}\left({}^{90}Y\right)\;\cdot \;C_{voxel}\left({}^{99m}Tc\right)}{C_{liver}\left({}^{99m}Tc\right)}$$

where *C*_*voxel*_(^99*m*^*Tc*) and *C*_*liver*_(^99*m*^*Tc*) are the ^99*m*^Tc-MAA SPECT count, in the voxel and the whole liver, respectively; and *A*_*liver*_(^90^*Y*) is the corresponding net injected activity of ^90^Y, in this case 2.9 GBq.

The absorbed dose map was calculated as the convolution of the voxel cumulated activity $\tilde{A}(r)$ and the VDK *K*(*r*)

(15) $$D(r)=\tilde{A}(r)*K(r)$$

(16) $$\;=1.443T_{1/2}\left({}^{90}Y\right)A_{voxel}*K(r)$$

where *T*_1/2_ is the ^90^*Y* half-life (64.2*h*)

The gamma index was used to compare the absorbed dose maps obtained by MC and DVK, defined as [59]

(17) $$\Gamma \left(r_{e},r_{R}\right)=\sqrt{\frac{\Delta r^{2}\left(r_{e},\;r_{R}\right)}{\delta r^{2}}+\frac{\Delta D^{2}\left(r_{e},\;r_{R}\right)}{\delta D^{2}}}$$

(18) $$\gamma \left(r_{R}\right)=\min\left\{\Gamma \left(r_{e},\;r_{R}\right)\right\}\;\forall r_{e}$$

where Δ*r*(*r*_*e*_*, r*_*R*_) is the distance between evaluated and reference point, Δ*D*(*r*_*e*_*, r*_*R*_) is the difference between doses at the evaluated and reference point, *δr* is the distance difference criterion, and *δD* is the dose difference criterion. The distance criterion was 3*mm*, the dose criterion was 3%, and the dose map calculated by MC was the reference and VDK the evaluation.

## 3 Result

### 3.1 Monoenergetic sDPK

**Fig. 2:**
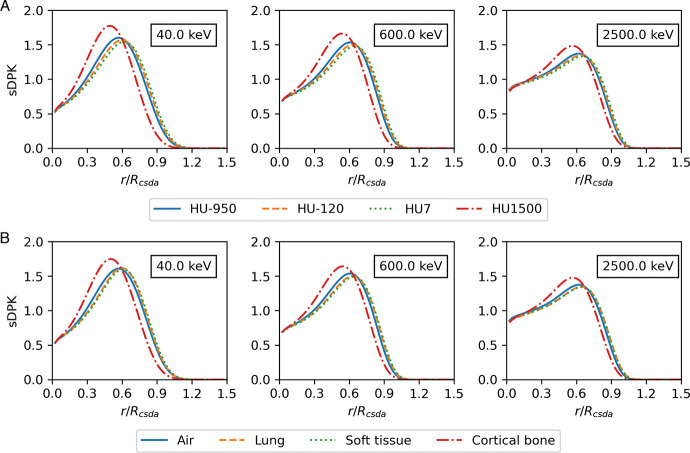
sDPK calculated by FLUKA MC for four materials from A) the training data set and B) the testing data set.

Figure 2 show the sDPK for a monoenergetic source of electrons obtained from calculating the energy deposited in each thickness shell at a distance by MC FLUKA and applying equation 7. The percentage error for each shell of sDPK was lower than 1%, a distance shorter than *R*_*CSDA*_ (*r* < *R*_*CSDA*_); when increasing the distance up to 1.2 · *R*_*CSDA*_, the percentage error increased by 4%. In some materials, it is found that the percentage error at distances larger than 1.2 · *R*_*CSDA*_ rises to 100%; therefore, longer distances have not been considered.

**Fig. 3:**
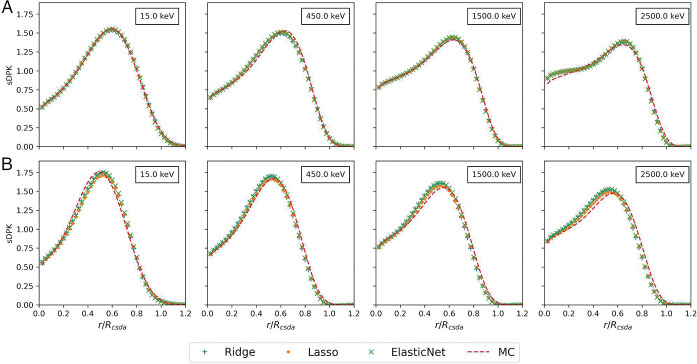
sDPK calculated by FLUKA MC and ML model for monoenergetic beta source in A) Lung, B) Compact Bone. The sDPK values are plotted as a function of the scaled distance.

Table 4 resumes results for two metrics considering evaluating the performance of different base regressors models. The coefficient of determination *R*^2^ reaches a value greater than 0.80 over the training set for the three base models. However, in the testing dataset, the maximum value of *R*^2^ achieved 0.76 for the Lasso base regressor. The lowest value was achieved for *RMSE* with RIDGE base regressor for training and Lasso base regressor for testing dataset. The sDPK estimated with the ML model for two materials of the testing set for four different monoenergetic electron sources are shown in Figure 3.

### 3.2 Beta-emitting radionuclide sDPK

The sDPK for ^89^Sr, ^90^Y, ^177^Lu, ^186^Re, and ^188^Re beta-emitting radionuclides were calculated. Emission spectra were taken from ICRP Publication 107 [60] and fitted with a smooth spline of degree 3 for each radionuclide. Then, 1000 values for pair (energy, probability) were calculated in the range of 10keV up to the maximum kinetic energy release for the radionuclide. The range of electrons for each material and energy was estimated using the analytic representation presented by Tabata et al. [49]. In the region where the ML model for calculating monoenergetic electron sDPK was valid (10*keV* to 3*MeV*), the analytical fit of the range showed a difference lower than 2% with NIST ESTAR [61].

**Fig. 4:**
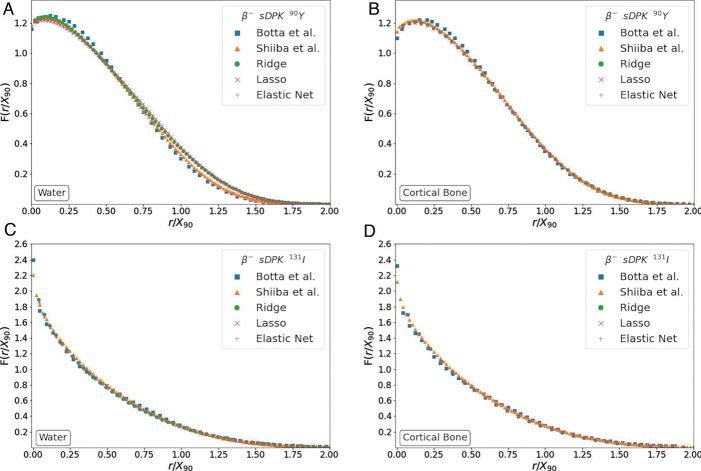
Benchmarking sDPK calculate by ML model with the sDPK reported by Botta et al. [55] and Shiiba et al. [56]

The ML model calculates monoenergetic sDPK for each energy, the material compound according to supplemental table 3, and the range for the material and energy considered. The beta-emitting radionuclide sDPK was determined using equation 6 considering a sphere of 120 shells and thickness of *r*_*N*_/100, where *r*_*N*_ was the range *R*_*CSDA*_ for the energy of maximum emission. Figure 4 shows beta-emitting radionuclide sDPK for a) water and b) compact bone, for ^90^Y and ^131^I, with the sDPK reported by Botta et al. [55] and Shiiba et al. [56]. It should be noted that the calculated sDPK was rescaled to the X90 scale [21]. This scale uses the distance at which 90% of the emitted energy is absorbed.

### 3.3 Application on dosimetry calculation

**Fig. 5:**
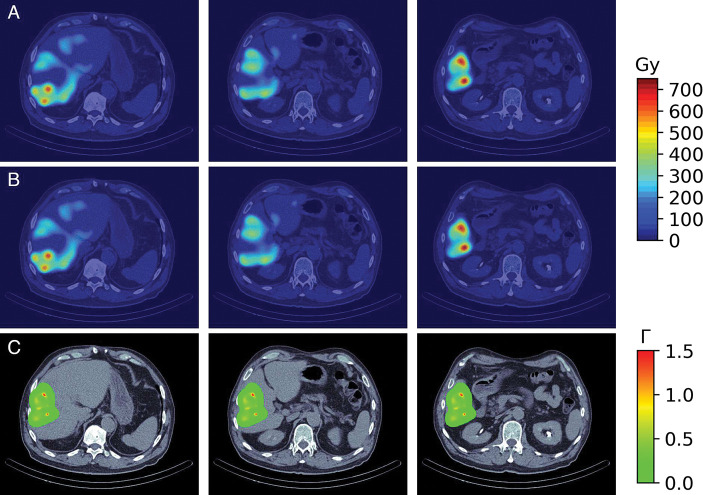
Results of dose absorbed calculate by A) MC and B) VDK. The figure C) is the Index gamma for the criterion 3*mm*/3%.

Figure 5 shows the absorbed dose map calculated by A) MC and B) VDK for the same three axial slices shown in Figure 1, and C) show the gamma index for the criterion 3*mm*/3%. The activity of each voxel was calculated by equation 14 and applied to equation 15. The voxelized kernel was calculated for each voxel where activity was greater than 0 using the calibration of Schneider et al. [40] to transform the Hu to the corresponding material compound. The sDPK for 90Y was calculated using the model with Lasso as regressor based.

The mean absorbed dose calculated by MC for the liver and the five VOIs considered is greater than the mean absorbed dose calculated by VDK by approximately 7%. The standard deviation means of absorbed dose at voxel levels calculated by FLUKA was less than 9% in the whole liver, and less than 1% in 5 VOIs considered. Table 5 summarizes the results obtained for the entire liver and the five VOIs.

In more than 94% of voxels, the gamma index was less than 1 for the 3*mm*/3% criterion in all regions considered. Figure 7 C) shows gamma index maps for the same three axial slices. As can be seen, at a zone of high dose the gamma index is greater than 1.

## 4 Discussion

The ML-based models with three different regressors based considered have been able to predict with reasonable accuracy the monoenergetic electron sDPK. The *R*^2^ for materials in the testing dataset was more significant than 0.75 in all models studied. The goodness or suitability of models decreases when the initial energy of the source increase (see Fig 3); this is most appreciated in air and lung. As radionuclides commonly used in nuclear medicine present electron emission with energies lower than 2.5*MeV* (e.g., ^90^Y = 2.28*MeV* and ^188^Re = 2.1*MeV*), the less performance at an energy greater than 2.5*MeV* has not a significant effect when calculating the sDPK for beta-emitting radionuclide. Furthermore, the probability of emission of electrons with energies higher than 2 MeV represents a small fraction compared to the probability of emission of electrons of lower energies where the models have good accuracy.

**Fig. 6:**
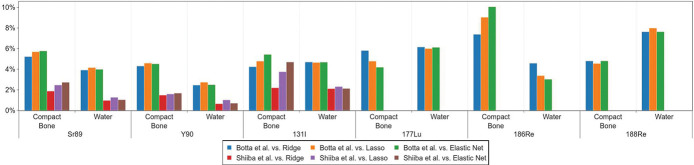
Mean absolute percentage error (MAPE) for water and compact bone, for all emitters compared to those published by Botta *et al.* [55] and Shiiba *et al.* [56].

Beta-emitting radionuclide sDPK calculated from monoenergetic sDPK estimated by ML models and using equation 6 showed a good correlation between values reported by Botta et al. and Shiiba et al. (see Fig 5). Figure 6 shows the MAPE for compact bone and water for all radionuclides, which was less than 10% compared to the sDPK reported. This discrepancy is mainly due to differences in the spectrum of emissions considered. The model for beta-emitting radionuclides sDPK does not consider the Auger and conversion electrons emitted in radionuclide decay, thus influencing at short-range level. As shown in figure 4, the non-negligible differences correspond to regions very close to the emission source.

The three ML-based models studied in this work have demonstrated the capability to quickly calculate the monoenergetic sDPK with the composition, density, energy, and range of material as input data. This allows the generation of monoenergetic sDPK easily for any material, and the beta-emitting radionuclide sDPK can be calculated by the model proposed for a wide range of radionuclides.

Finally, the application of sDPK for dosimetric calculation showed a good agreement with FLUKA MC calculation of maps of absorbed dose. Index gamma was less than 1 in more than 94% of voxels. However, voxel convolution underestimated the absorbed dose by 6% approximately. The model proposed to estimate the absorbed dose by voxels kernel convolution has shown a real advantage over FLUKA MC when comparing the required calculation time, i.e. 7 minutes versus 40 hours, respectively. Moreover, the capability of ML-based model’s to quickly calculate several monoenergetic electron sDPK was demonstrated, which were further used to estimate the beta-emitting radionuclide sDPK tailored to each material.

## 5 Conclusion

An ML model was developed to show the capacity to accurately calculate the sDPK for monoenergetic beta sources in a wide range of energy and materials compounds. Also, a model for calculating the sDPK for beta-emitting radionuclide from the monoenergetic sDPK allows obtaining the VDK for calculating the absorbed dose at a patient-specific level in a short time.

## Figures and Tables

**Table 1:** Example of a lengthy table which is set to full textwidth

Radionuclide	Half-life [days]	*E_avg_*(*E_max_*)[keV]	Therapeutic indication

^89^Sr	50.6	587.1 (1502.2)	Relief of pain skeletal metastases
^90^Y	2.7	932.4 (2275.6)	Hepatocellular cancer and liver metastasis Non-Hodgkin’s lymphoma
^131^I	8.0	191.6 (806.9)	Hyperthyroidism, differentiated thyroid cancer Non-Hodgkin’s lymphoma
^177^Lu	6.7	148.8 (496.8)	Neuroendocrine tumors Prostate tumors
^186^Re	3.7	359.2 (1072.7)	Relief of pain, skeletal metastases
^188^Re	16.7	795.4 (2120.4)	Relief of pain, skeletal metastases

**Table 2:** Material composition in weight fraction and density [*g*/*cm*^3^] of training dataset

Material	H	C	N	O	Na	Mg	P	S	Cl	Ar	K	Ca	Density

HU-950			0.755	0.232						0.013			0.0279
HU-120	0.103	0.105	0.031	0.749	0.002		0.002	0.003	0.003		0.002		0.4810
HU-83	0.116	0.681	0.002	0.198	0.001			0.001	0.001				0.9572
HU-53	0.113	0.567	0.009	0.308	0.001			0.001	0.001				0.9581
HU7	0.108	0.356	0.022	0.509	0.000		0.001	0.002	0.002				1.0108
HU18	0.106	0.284	0.026	0.578	0.000		0.001	0.002	0.002		0.001		1.0030
HU80	0.103	0.134	0.030	0.723	0.002		0.002	0.002	0.002		0.002		1.0591
HU120	0.094	0.207	0.062	0.622	0.006		0.000	0.006	0.003		0.000		1.1187
HU200	0.095	0.455	0.025	0.355	0.001		0.021	0.001	0.001		0.001	0.045	1.1111
HU300	0.089	0.423	0.027	0.363	0.001		0.030	0.001	0.001		0.001	0.064	1.1644
HU400	0.082	0.391	0.029	0.372	0.001		0.039	0.001	0.001		0.001	0.083	1.2236
HU500	0.076	0.361	0.030	0.380	0.001	0.001	0.047	0.002	0.001			0.101	1.2828
HU600	0.071	0.335	0.032	0.387	0.001	0.001	0.054	0.002				0.117	1.3420
HU700	0.066	0.310	0.033	0.394	0.001	0.001	0.061	0.002				0.132	1.4012
HU800	0.061	0.287	0.035	0.400	0.001	0.001	0.067	0.002				0.146	1.4604
HU900	0.056	0.265	0.036	0.405	0.001	0.002	0.073	0.003				0.159	1.5196
HU1000	0.052	0.246	0.037	0.411	0.001	0.002	0.078	0.003				0.170	1.5788
HU1100	0.049	0.227	0.038	0.416	0.001	0.002	0.083	0.003				0.181	1.6380
HU1200	0.045	0.210	0.039	0.420	0.001	0.002	0.088	0.003				0.192	1.6972
HU1300	0.042	0.194	0.040	0.425	0.001	0.002	0.092	0.003				0.201	1.7564
HU1400	0.039	0.179	0.041	0.429	0.001	0.002	0.096	0.003				0.210	1.8156
HU1500	0.036	0.165	0.042	0.432	0.001	0.002	0.100	0.003				0.219	1.8748
HU1600	0.034	0.155	0.042	0.435	0.001	0.002	0.103	0.003				0.225	1.9340

**Table 3:** Material composition in weight fraction and density [*g*/*cm*^3^] of testing dataset

Material	H	C	N	O	Na	Mg	P	S	Cl	Ar	K	Ca	Density

Air		0.0001	0.755	0.231						0.012			0.0012
Lung	0.101	0.102	0.028	0.757	0.001	0.0007	0.0008	0.002	0.002		0.001	0.00009	1.05
Soft Tissue	0.104	0.232	0.024	0.630	0.001	0.0001	0.001	0.002	0.001		0.002	0.0002	1.00
Cortical Bone	0.047	0.144	0.042	0.446		0.002	0.105	0.003				0.209	1.85

**Table 4:** Results obtained for two metrics applied *R*^2^ and RMSE, for the three regressors consider

	Training		Testing	

	*R* ^2^	RMSE	*R* ^2^	RMSE
Ridge	**0.850(0.143)**	**0.0313(0.0144)**	0.753(0.180)	0.0385(0.0161)
Lasso	0.839(0.149)	0.0326(0.0146)	**0.766(0.184)**	**0.0367(0.0149)**
ElasticNet	0.838(0.149)	0.0329(0.0149)	0.765(0.182)	0.0369(0.0148)

**Table 5:** Dose absorbed calculated by MC and DVK expressed as $\bar{D}\pm \sigma$ and [*D_min_*, *D_max_*]. Also, the gamma index calculated is reported for the Liver, and five VOIs consider.

Region	Dose by MC [*Gy*]	Dose by DVK [*Gy*]	Gamma Index

Liver	88.87 ± 124.76 [0.00, 728.70]	82.02 ± 116.57 [0.00, 676.23]	94.96 %
VOI 1	387.61 ± 142.95 [25.62, 728.7]	361.77 ± 134.08 [20.33, 676.23]	98.19%
VOI 2	329.89 ± 148.59 [2.24, 673.77]	307.61 ± 139.29 [1.52, 627.85]	96.01%
VOI 3	334.85 ± 83.3 [42.9, 645.47]	312.69 ± 77.41 [40.23, 599.25]	94.82%
VOI 4	265.95 ± 47.29 [114.11, 398.24]	248.45 ± 44.17 [106.17, 371.52]	90.02%
VOI 5	205.84 ± 41.99 [43.75, 297.07]	192.52 ± 39.2 [40.68, 278.4]	96.08%

## Data Availability

Anonymized ^99*m*^Tc-MAA SPECT/CT DICOM data including segmented lesions for select patients are available at the University of Michigan Library Deep Blue repository: 10.7302/v07v-z854 and 10.7302/pf4m-vn04 The datasets generated during and/or analysed during the current study are available from the corresponding author on reasonable request.
